# Characteristics and risk factors for microbial infections during cancer immune checkpoint therapy

**DOI:** 10.1002/cam4.3532

**Published:** 2020-11-07

**Authors:** Yada Kanjanapan, Desmond Yip

**Affiliations:** ^1^ Department of Medical Oncology The Canberra Hospital Garran Australia; ^2^ ANU Medical School Australian National University Canberra Australia

**Keywords:** cancer, checkpoint inhibitor, Immunotherapy, infection, microbes

## Abstract

The risk of infection in patients receiving immune checkpoint inhibitor (ICI) therapy is not well understood. Immune‐related adverse events requiring immunosuppressive therapy may impact infection risk. ICIs may induce an exaggerated immune response to latent infection. We assessed the incidence and risk factors for infections during cancer ICI therapy. A retrospective chart review of solid tumor patients treated with ICIs was conducted. Infectious episodes were defined as those where a microbial organism was cultured or identified through polymerase chain reaction. Infections which occurred during and up to 1 year following ICI therapy were considered “post‐ICI” infections. Of 327 patients, 47% had melanoma and 36% had non‐small cell lung cancer. The majority (77%) received single agent anti‐PD(L)1 antibody, 14% received combination anti‐PD(L)1 and anti‐CTLA4 antibody, and 9% single agent anti‐CTLA4 antibody. Infections occurred in 89 (27%) in the post‐ICI compared with 111 (34%) patients in the pre‐ICI period (*p* = 0.57). The most common types of infection were respiratory, genitourinary, and cutaneous infections. On multivariate analysis, only age over 67 years significantly predicted for development of infection on ICI (HR 1.73, *p* = 0.04). We did not find receipt of corticosteroids, combination ICI therapy, diabetes, or gender to significantly impact on infection risk. The rate of microbial infections among solid tumor patients receiving ICI therapy was 27%, comparable to the infection rate of 34% in the same cohort of patients in the period pre‐ICI therapy. Age over 67 years was significantly associated with infection post‐ICI.

## Graphical abstract

There was a 27% rate of infection among solid tumor patients on immune checkpoint inhibitors, which is not increased from the baseline infection rate. Age over 67 years was significantly associated with risk of developing an infection during cancer immunotherapy.

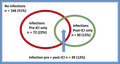

## 1 INTRODUCTION

The impact of immunotherapy on the risk of microbial infection has not been well established in the literature. The immune system is the common defense mechanism against cancer and pathogenic microorganisms. While immune checkpoint inhibitor (ICI) therapy stimulates the immune system's anticancer effect, any interplay with response to microbial infection remains unclear. Furthermore, patients treated with immunotherapy have a risk of developing immune‐related adverse events requiring prolonged courses of corticosteroids, with or without additional immunosuppressive therapy (e.g. infliximab), which may modulate patients’ infection risk during this period.

While prospective clinical trials of immunotherapy in various cancers have not reported an increased infection risk, it is not known if there may be under‐reporting by investigators (as causality to the immunotherapy is not clearly established); and details on the microbial composition are not fully captured. The KEYNOTE‐010 trial reported pneumonia (any grade 1.5%, 0.9% grade 3‐5), lung infection (0.3%), oral candidiasis (0.3%), and urinary tract infection (0.3%) occurring in non‐small cell lung cancer (NSCLC) patients treated with pembrolizumab.^1^ In a study of nivolumab in NSCLC there was a case of pneumonia and one case of grade 3‐4 herpes zoster infection.^2^

A hyper‐response to microorganisms in the setting of programmed death‐1 (PD‐1) or programmed death ligand‐1 (PD‐L1) pathway inhibition has been postulated to account for observations of acute pulmonary tuberculosis reactivation on ICI therapy.^3^, ^4^, ^5^, ^6^, ^7^ These patients developed culture positive Mycobacterium tuberculosis following 5‐8 cycles of anti‐PD1 ICI, in the absence of any immune‐related adverse events nor any concomitant immunosuppressive medication.^3^, ^4^ A patient with latent tuberculosis presenting with tuberculous pericarditis following anti‐PD1 antibody therapy has also been described.^8^ In this paper, we examine the incidence and risk factors for microbial infections in solid tumor patients receiving ICI therapy.

## 2 METHODS

The study included solid tumor patients who received ICI therapy (anti‐PD1, anti‐PDL1 and/or anti‐CTLA4 antibodies) at The Canberra Hospital, Australian Capital Territory (ACT), Australia. The Department of Medical Oncology electronic prescribing system CHARM (CHARM Health, Brisbane) was used for case identification. Consecutive patients who commenced ICI therapy between Nov 2012 and April 2019 were included. These ICI therapies included ipilimumab, nivolumab, pembrolizumab, atezolizumab, and durvalumab which were dosed as per weight based dosing as per their product information at the time. There was no dose modification for treatment‐related toxicity, with treatment interruption and cessation for high grade toxicities, consistent with standard practice.

A retrospective chart review was conducted to collect clinicopathological characteristics, treatment, and outcome data. For each identified patient case, the hospital electronic medical records system was examined for any positive microbiology results for that patient. In the study location (Canberra), there is one public pathology service (ACT Pathology), which processes microbiology results for all inpatients at The Canberra Hospital, and additionally services a significant proportion of outpatient requests. While some outpatient testing may have occurred in private pathology labs (e.g. referred through the general practitioner), interrogation of the public pathology service is expected to capture the majority of infectious episodes.

Infectious episodes were defined as those where a microbial organism was identified (through culture or polymerase chain reaction). Infections occurring during and up to 1 year following ICI therapy were classified as “post‐ICI” infections, and infections prior to ICI therapy commencement as “pre‐ICI” infections. Positive cultures from surveillance swabs (such as for methicillin‐resistant Staphylococcus aureus [MRSA]) or where the microbiology report deemed a likely contaminant organism had been isolated were excluded in our analysis. This study received institutional ethics approval through the ACT Health Human Research Ethics Committee (Approval number 2019/LRE/00052).

### 2.1 Statistical methods

The association between infection and categorical variables (gender, diabetes, corticosteroid‐use) was assessed using Fisher's exact test. The association between infection and continuous variables (age) was tested using the Mann‐Whitney test. We compared the differences in the rates of infection in the pre‐ICI and post‐ICI periods using the McNemar test. Statistical significance was set at the 0.05 level. Statistical analyses were performed using IBM SPSS Statistics for Windows, version 26.0 (SPSS Inc.).

## 3 RESULTS

### 3.1 Patient characteristics

A total of 327 patients with solid tumors were treated with ICI therapy (Table 1). Their median age was 67 years and 60% were male. The most common tumor types were cutaneous melanoma (47%) and NSCLC (36%) with other tumor types (genitourinary, head and neck, gynecological, breast cancer, mesothelioma, non‐cutaneous melanoma, sarcoma, and Merkel cell carcinoma) each representing <10% of the study population.

**TABLE 1:** Baseline characteristics (n = 327)

Characteristics	Category	Infection post‐ICI (n = 89)	No infection post‐ICI (n = 238)	*p*‐value
Age	Median (range)	69 (24‐94)	66 (26‐93)	0.07
Gender	Male	57 (64%)	138 (58%)	0.38
	Female	32 (37%)	100 (42%)	
Tumor type	Melanoma (cutaneous)	36 (40%)	119 (50%)	0.03
	Lung (non‐small cell)	32 (36%)	87 (37%)	
	Genitourinary	14 (16%)	15 (6%)	
	Gynecological	3 (3%)	1 (<1%)	
	Head and neck	3 (3%)	3 (1%)	
	Non‐cutaneous melanoma	1 (1%)	2 (1%)	
	Breast	0 (0%)	3 (1%)	
	Merkel cell carcinoma	0 (0%)	2 (1%)	
	Mesothelioma	0 (0%)	3 (1%)	
	Sarcoma	0 (0%)	3 (1%)	
Treatment stage	Localized Disease	4 (4%)	16 (7%)	0.61
	Metastatic Disease	85 (96%)	222 (93%)	
Treatment type	Anti‐PD(L)1	74 (83%)	179 (75%)	0.48
	Anti‐CTLA4	5 (6%)	23 (10%)	
	Anti‐PD(L)1 + antiCTLA4	10 (11%)	36 (15%)	
Treatment duration	Cycles received	7 (1‐98)	5 (1‐71)	0.38
Corticosteroid	Corticosteroid for immune‐related adverse events	46 (52%)	103 (43%)	0.21
Comorbidities	Diabetes	6 (7%)	29 (12%)	0.23
	Charlson comorbidity index^(9)^ ‐ median (range)	9 (4‐14)	9 (2‐15)	0.09

### 3.2 Characteristics of infections

**FIGURE 1:**
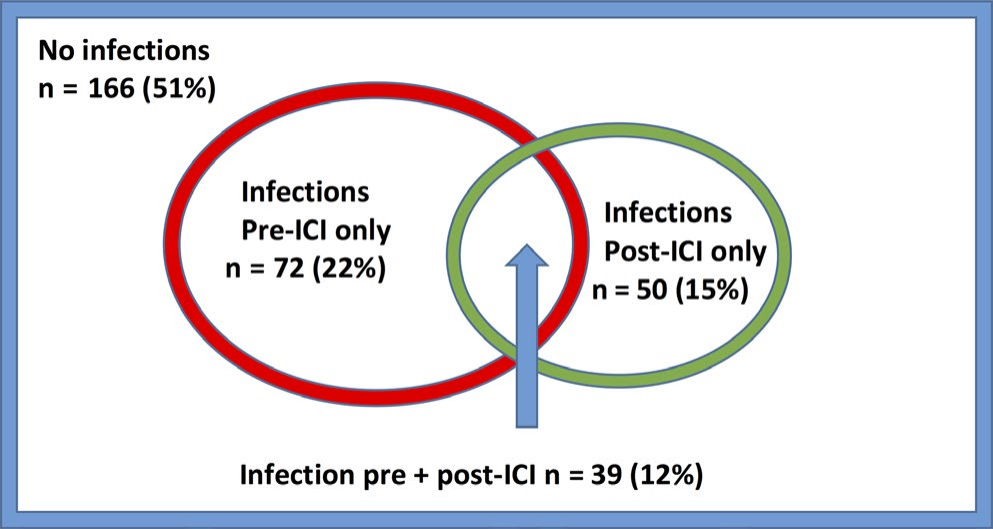
Distribution of patients with and without infection

A total of 535 infectious episodes were cataloged (Table 2). The distributions in types of infections were similar in the pre‐ICI and post‐ICI periods. The commonest organ systems involved were cutaneous, respiratory, genitourinary, and bacteremia. Most infections were caused by bacteria, with fungal and viral infectious agents also represented. Infections occurred in 111 (34%) of patients in the pre‐ICI compared 89 (27%) in the post‐ICI period (*p* = 0.57) (Figure 1). Of patients with post‐ICI infections, 51/89 (57%) had Common Terminology Criteria for Adverse Events (CTCAE) Grade 3/4 infections, requiring intravenous antimicrobial therapy and/or hospitalization.

**TABLE 2:** Infectious organisms cultured pre‐ and post‐immune checkpoint inhibitor.

Infection type	Organism	Number of cases	
		Pre‐Immunotherapy	Post‐immunotherapy
Cutaneous		109 (32%)	47 (24%)
Bacterial	*Staphylococcus aureus*	55	18
	Mixed anaerobic bacteria	12	9
	*Enterobacte*r species	6	3
	*Pseudomonas aeruginosa*	3	3
	*Streptococcus* species	3	1
	*Escherichia coli*	2	0
Fungal	*Candida* species	7	5
Viral	HSV/VZV	6	4
Genitourinary		100 (30%)	66 (33%)
Bacterial	*Escherichia coli*	56	31
	*Klebsiella* species	12	8
	*Enterococcus faecalis/faecium*	6	4
	*Streptococcus* species	6	0
	*Citrobacter* species	3	2
	*Morganella morganii*	3	0
	*Pseudomonas aeruginosa*	2	3
	Mixed anaerobic bacteria	2	0
	*Proteus mirabilis*	1	3
Fungal	*Candida* species	5	9
Respiratory		86 (26%)	57 (29%)
Bacterial	*Staphylococcus aureus*	13	11
	Mixed anaerobic bacteria	13	5
	*Haemophilus influenzae*	10	5
	*Enterobacter* species	7	1
	*Streptococcus pneumoniae*	4	2
	*Klebsiella pneumoniae/oxytoca*	4	0
	*Pseudomonas aeruginosa*	2	5
	PJP	2	3
	*Escherichia coli*	1	4
	*Moraxella catarrhalis*	1	1
Fungal	*Candida albicans*	10	12
	*Aspergillus* species	2	1
Viral	RSV, HMV, Rhinovirus, Adenovirus	4	3
	HSV	2	0
Bacteremia		19 (6%)	18 (9%)
	*Escherichia coli*	6	5
	*Staphylococcus* aureus	2	3
	Other *staphylococcus* species	4	1
	*Klebsiella pneumoniae*	1	0
	*Streptococcus* species	1	3
	*Bacteroides fragilis*	1	0
	*Citrobacter freundii*	1	0
	*Proteus* species	0	2
	*Enterococcus faecalis*	0	1
	*Pseudomonas aeruginosa*	0	1
Gastrointestinal		12 (4%)	8 (4%)
*Bacterial*	*Campylobacter species*	2	1
	*Escherichia coli*	1	1
	*Salmonella typhimurium*	1	0
	*Enterococcus faecium*	0	1
	*Pseudomonas aeruginosa*	0	1
*Fungal*	*Candida* species	3	1
	*Aspergillus fumigatus*	1	0
*Viral*	CMV	0	0
	HSV1	0	1
Bone		6 (2%)	1 (1%)
*Bacterial*	*Escherichia coli*	2	0
	*Staphylococcus aureus*	1	1
	*Staphylococcus epidermidis*	1	0
	*Viridans streptococcus*	1	0
	Mixed anaerobic bacteria	1	0
Ocular/CNS		5 (1%)	1 (1%)
*Bacterial*	*Staphylococcus aureus*	2	0
	*Corynebacterium macginleyi*	1	0
	*Moraxella* species	1	0
	*Staphylococcus epidermidis*	1	0
*Viral*	HSV1	0	1
Total		337 (100%)	198 (100%)

Note

Representative examples within each organ‐site category are given, but the table does not display the complete list of microorganisms cultured.

Abbreviations: CNS, central nervous system; HMV, human metapneumovirus; HSV, herpes simplex virus; PJP, *Pneumocystis jiroveci* pneumonia; VZV, varicella zoster virus.

### 3.3 Timing of infections

**FIGURE 2:**
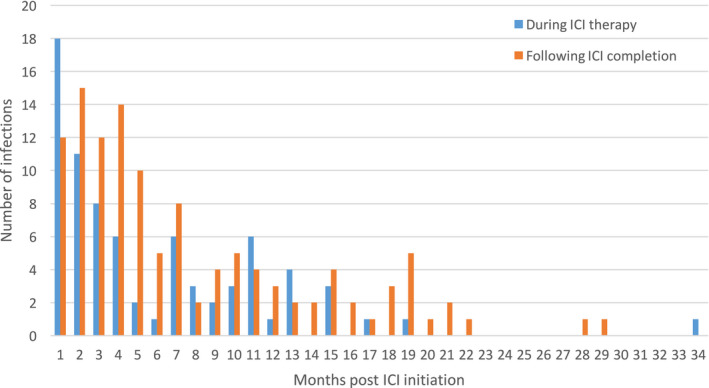
Timing of infections following ICI therapy

The majority of post‐ICI infections occurred within 12 months of ICI commencement, with the median time to onset of infection being 123 days (range 1‐940 days). All except three episodes of infection occurred within 24 months post‐ICI initiation (Figure 2). Infections occurred during the course of ICI in 77/196 (39%) of cases, and following cessation of ICI in 119/196 (61%) of cases.

### 3.4 Risk factors for infections

The risk for microbial infection during ICI‐therapy was increased with age over 67 years on univariate and multivariate analyses (hazard ratio [HR] 1.73, 95% confidence interval [CI] 1.04‐2.87, *p* = 0.04) (Table 3). Although a diagnosis of diabetes had a hazard ratio of 0.44 for infection, this association was not statistically significant (*p* = 0.09). The patient's Charlson comorbidity index^9^ score was not associated with infection risk on univariate analysis. This variable was excluded from multivariate analysis given the score included age and diabetes, which overlapped with two of the variables examined in the multivariate model. Neither gender nor the use of combination immunotherapy over single‐agent ICI had a statistically significant impact on infection risk.

**TABLE 3:** Predictors for microbial infection on ICI

Predictor	Univariate		Multivariate	
	HR (95% CI)	*p*‐value	HR (95% CI)	*p*‐value
Age (>67 years)	1.76 (1.07‐2.89)	0.03	1.73 (1.04‐2.87)	0.04
Gender (female)	0.78 (0.47‐1.28)	0.32	0.84 (0.50‐1.40)	0.50
Diabetes	0.52 (0.21‐1.30)	0.16	0.44 (0.12‐1.13)	0.09
Corticosteroids	1.40 (0.86‐2.29)	0.18	1.51 (0.91‐2.49)	0.11
Combination immunotherapy	0.64 (0.31‐1.30)	0.22	0.61 (0.29‐1.28)	0.19

Receipt of corticosteroid was not associated with a statistically significant increased risk of microbial infection (HR 1.51, 95% CI 0.91‐2.49, *p* = 0.11). Seven patients received infliximab in addition to corticosteroids for immune‐medicated colitis. Three of these patients did not experience any infections. There were two patients with infections only post‐ICI with infliximab therapy, and two patients with infections only pre‐ICI. The infectious episodes post‐ICI in infliximab‐treated patients included urinary tract infection (*Citrobacter koseri*, *Klebsiella pneumoniae*, and *Proteus vulgaris*) and bacteremia (*Proteus penneri*).

## 4 DISCUSSION

Our cohort of 327 solid tumor patients treated with ICI had a microbial infection rate of 27%. A series of 167 NSCLC patients from two centers in Kyoto, Japan, reported 19.2% experienced infectious diseases.^10^ This is closer to the rate of 16% (51/327), when considering only grade 3/4 infections post‐ICI in our series. Another retrospective series from the Memorial Sloan Kettering Cancer Centre (MSKCC) of 740 metastatic melanoma patients treated with ICI reported serious infections in 7.3% of patients.^11^ The differences in infection incidences among the three studies are most likely attributable to varying definitions applied for infections. The Kyoto study defined an infectious disease as an infection requiring administration of an antimicrobial agent, which occurred any time from initiation of ICI to 3 months after discontinuation.^10^ The MSKCC series examined serious infections which required hospitalization or parenteral antimicrobials, from ICI initiation until 1‐year post‐discontinuation.^11^

Of note, the majority of patients in the MSKCC series^11^ received the anti‐CTLA4 antibody ipilimumab (73%), compared with 9% in our study; and the Kyoto cohort^10^ consisted entirely of patients treated with the anti‐PD1 antibody nivolumab. Whether the class of ICI therapy affects infection risk is not established, and the sample size of available studies including ours limit the ability to assess this. We explored any impact of combination versus single‐agent immunotherapy on infection risk. This is based on the hypothesis that combination immunotherapy has a higher incidence of immune‐related adverse events, resulting in the need for treatment with corticosteroid and/or other immunosuppressive agents,^12^ that ultimately predispose patients to developing microbial infections. However, we found no statistically significant impact of combination ICI therapy on infection risk on univariate or multivariate analysis. Although the use of immunosuppressive agents such as infliximab can predispose patients to opportunistic infections such as *Pneumocystis jiroveci* pneumonia (PJP), routine cotrimoxazole prophylaxis with prolonged corticosteroid therapy at our institution may have mitigated this risk.

To date, our study is unique in examining microbial infection rate both prior to as well as during ICI therapy. The method employs each patient as his or her own control, in comparing their rate of infection with and without the impact of ICI. This puts into perspective the infection rate during ICI; compared with the expected infection rate in the same patient population. Of note, some patients received chemotherapy prior to ICI (subgroup of NSCLC patients were treated with second line nivolumab in this setting), and the impairment in immune recovery following chemotherapy is a potential confounder in assessment of the pre‐ICI infection rate.^13^ With the above limitation in mind, our findings provide evidence that the rate of microbial infection was not significantly increased during ICI therapy; with an infection rate of 27% post‐ICI compared with 34% pre‐ICI.

The median onset of infection from ICI initiation was 123 days in our study, comparable with 135 days in the MSKCC series,^11^ but later than 90.3 days in the Kyoto cohort.^10^ Of note, there was a wide range in timing of infection onset, with a range of 1 to 940 days in our series, and 6‐491 days in the MSKCC study. ICIs have relative long half‐lives (pembrolizumab—22 days; nivolumab—25 days)^14^ and resultant immunomodulatory effects are expected to persist beyond the time of drug administration. Furthermore, many immune‐mediated adverse events have a delayed onset of months following ICI initiation^15^, ^16^; thus, the use of corticosteroids or other immunosuppressive for toxicity treatment, and secondary opportunistic infections can have delayed onset. We found a lack of infections occurring beyond 2 years (>730 days) post‐ICI initiation, however, this observation maybe biased by fewer cases remaining for follow‐up beyond 2 years. In our series, the median duration of treatment was 84 days and median follow‐up was 273 days.

The most common types of infection in our series were respiratory, genitourinary, and cutaneous infections, in both the pre‐ICI and post‐ICI time periods. The majority of infections were bacterial; although fungal and viral infections were also represented. The proportional distribution of infectious organisms is similar to other series. The MSKCC group reports a predominance of bacterial infections, mostly of pulmonary or bloodstream source.^11^ The Kyoto group also found the commonest type of infection to be bacterial pneumonia followed by bacteremia.^10^ Importantly, opportunistic infections such as PJP were encountered but not over‐represented in the post‐ICI period from our series.

Reactivation of latent infection, including tuberculous and viral reactivations, are of interest in the context of ICI therapy. There are case series reporting on acute tuberculosis in cancer patients treated with anti‐PD1 antibodies,^3^, ^4^, ^6^, ^7^, ^8^ although the exact pathophysiologic mechanism has not been elucidated. A favored hypothesis is an immune reconstitution inflammatory syndrome‐like phenomenon, whereby ICI therapy potentiates activity of tuberculosis‐specific T cells.^7^ The observation that cases in the literature developed clinically evident tuberculosis within 3 months of ICI inferred reactivation of latent tuberculosis, rather than acute infection.^7^ Interestingly, none of the cases reported to date received corticosteroid therapy for immune‐related adverse events. Hypersensitivity response was thought to be the mechanism behind a case of tuberculous pericarditis developing 3 months following nivolumab for metastatic lung adenocarcinoma.^8^ In all cases, antituberculosis therapy was effective in controlling infection, and the majority reported successful continuation of cancer ICI therapy. With this background, we particularly examined for, and found no excess of tuberculosis reactivation in our series. A likely explanation is our patient demographic has a relatively low level of tuberculosis infection compared with endemic regions.

In our study, the risk of infection on immunotherapy was increased in patients aged 67 and greater. Immunosenescence, the decrease in immune function with age, is a recognized phenomenon.^17^ Potential underlying contributing factors include thymic involution, chronic antigen stimulation, signal transduction changes in immune cells, and protein‐energy malnutrition.^17^ This supports the hypothesis that age directly impacts on infection risk, as opposed to any modulation from ICI exposure. Interestingly, the Charlson comorbidity index which incorporates age, was not associated with infection risk; suggesting that age is the strongest independent predictive factor.

Fujita et al., reported an increased risk of infection on ICI in NSCLC patients with concomitant diabetes with an odds ratio of 3.61.^10^ Contemporary studies have demonstrated an increased risk for infectious diseases and worse outcome from infections in patients with diabetes.^18^, ^19^ The risk is particularly apparent for bacterial infections, attributable to a dysregulated immune system.^20^ In our study, although patients with diabetes had a hazard ratio of 0.44 for infection; the confidence interval crossed one (95% CI 0.12‐1.13) with an insignificant p‐value of 0.09, implying there is no statistically significant difference in infection rate in patients with or without diabetes. Furthermore, Del Castillo et al. previously showed receipt of corticosteroids to be associated with increased risk of serious infections in melanoma patients receiving ICI.^11^ We were unable to definitively validate the associations between infections on ICI with diabetes and corticosteroids seen in two previous series, which may be due to the limitation of our sample size.

There are limitations to our study. Due to its retrospective nature, it is not possible to compare outcomes in a control group of patients with balanced baseline characteristics. We attempted to overcome this by using each individual patient as his/her own control, in comparing occurrence of microbial infection in the period pre‐ICI and post‐ICI. The major limitation of this approach is the difference in duration of follow‐up in the pre‐ICI and post‐ICI periods. Furthermore, we acknowledge that there is a period of impaired immune recovery following cytotoxic cancer therapy, relevant in some cases, for example, NSCLC patients receiving ICI as second line therapy.^13^ We defined an infectious episode to be one where there was positive microbial culture. This would not capture infectious episodes where there was no microbiological confirmation; such as pneumonia based on clinical and radiological diagnosis alone, or where antimicrobial therapy was initiated prior to cultures being taken. We also acknowledge potential inclusion of clinically insignificant microbial cultures, including commensal organisms or contaminants; although these are excluded where identified on the microbiological reports.

We found 27% of solid cancer patients developed culture‐positive infections during and up to 1 year following ICI therapy. This rate was comparable to a 34% incidence of infections in the period prior ICI therapy. The site of infection and type of infectious organism were also similar in the pre‐ and post‐ICI periods. Age over 67 years was the only factor significantly associated with development of microbial infection. Diabetes and recipient of corticosteroids did not influence the infection risk, although this may be partially due to antimicrobial prophylaxis for opportunistic infections in patients requiring prolonged corticosteroid administration.

## CONFLICT OF INTEREST

All authors indicate that they do not have any conflicts of interest with regards to this work.

## AUTHOR CONTRIBUTIONS

Yada Kanjanapan: Conceptualization, methodology, data curation, formal analysis, writing‐ original draft, and editing. Desmond Yip: methodology, writing‐review, and editing.

## Data Availability

The data that support the findings of this study are available on request from the corresponding author. The data are not publicly available due to privacy or ethical restrictions. This study received institutional ethics approval through the Australian Capital Territory Health Human Research Ethics Committee (ACT HREC 2019/LRE/00052).
